# Supplementary material for: Physiologically Based Biopharmaceutics Modeling of Food Effect for Basmisanil: A Retrospective Case Study of the Utility for Formulation Bridging
Source: Pharmaceutics. 2023 Jan 5;15(1):191. doi: 10.3390/pharmaceutics15010191 (PMC9862143; doi:10.3390/pharmaceutics15010191)
Supplement: Supplementary file 1 [file pharmaceutics-15-00191-s001.zip › pharmaceutics-2101046-supplementary.pdf]

# Supplementary Material

**Fig.S1.** Decision tree for food effect model optimization (Riedmaier et al.)

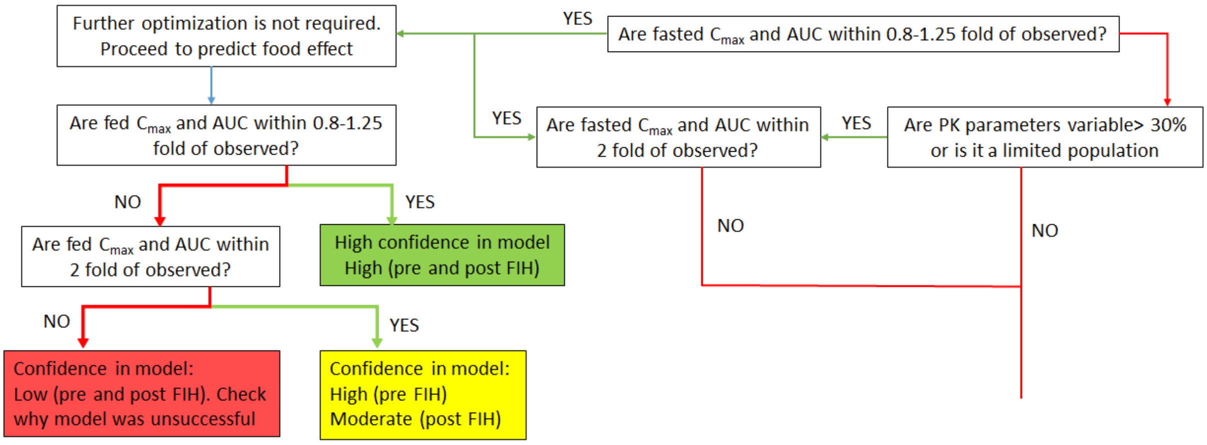

**Fig.S2**. Parameter sensitivity analysis for 120 mg granules under simulated fasted and fed states a) bile salt solubilization ratio b) permeability c) particle size d) stomach pH

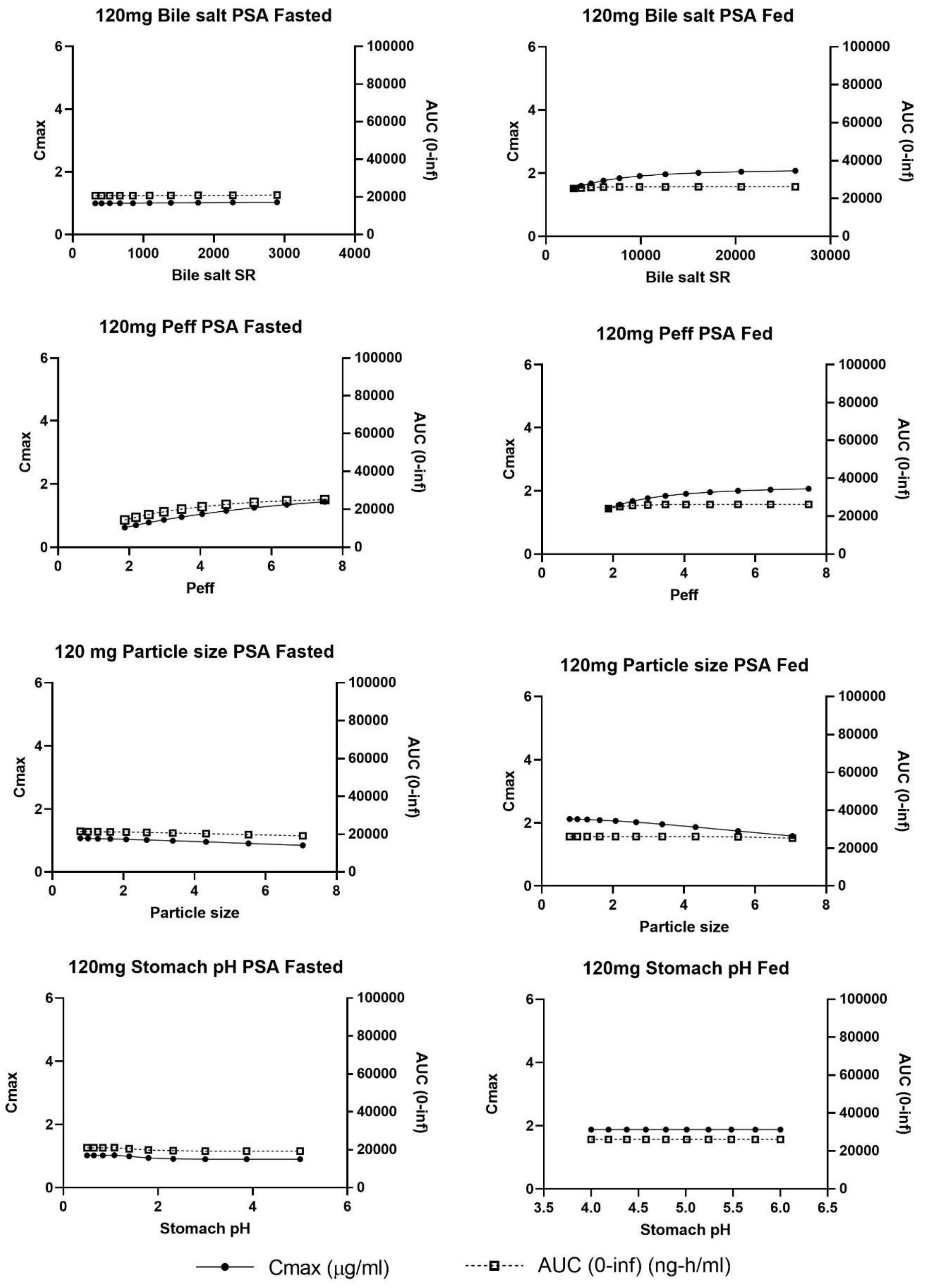

**Table.S1.** Tabular summary of parameter sensitivity analysis for 120 mg granules in sachet

| Parameters | Fasted State | | | Fed State | | |
|---|---|---|---|---|---|---|
| | Limits | Cmax (ng/ml) | AUC(0-inf) (ng.h.ml) | Limits | Cmax (ng/ml) | AUC(0-inf) (ng.h.ml) |
| **Peff** | 1.87-7.5 **(3.75)** | 62-143 | 69.3-121 | 1.87-7.5 **(3.75)** | 77.4-110 | 91.9-100 |
| **Particle size (D50)** | 0.78-7.05 **(2.35)** | 107-84.4 | 103-92.5 | 0.78-7.05 **(2.35)** | 113-84.6 | 100-97.3 |
| **Bile salt Solubiliza-tion Ratio** | 323-2897.1 **(965.7)** | 99.7-103 | 99.7-101 | 2917-26300 **(8753.6)** | 81.3-110 | 96.9-100 |
| **Stomach pH** | 0.5-5 **(1.3)** | 101-89.5 | 101-92.9 | 4-6 **(4.9)** | 100-100 | 100-100 |

Limits: Lower limit- upper limit (baseline value),
Cmax and AUC calculated as deviation from baseline value within 80-125%, beyond 80-125%

**Table. S2.** Experimental values of water solubility of compounds along with GastroPlus™ predictions.

| Compound | Molecular Weight (grams/mol) | Melting point (°C) | Prediction temperature (°C) | Experimental Solubility (g/L) | GastroPlus predicted solubility (g/L) | Reference |
|---|---|---|---|---|---|---|
| Paracetamol | 151.17 | 170.05 | 36.85 | 27.33 | 34.84 | [1] |
| Allopurinol | 136.11 | 362.35 | 36.85 | 0.42 | 0.43 | [1] |
| Budesonide | 430.54 | 260.85 | 36.85 | $24.13x10^{-3}$ | $65.14x10^{-3}$ | [1] |
| Furosemide | 330.74 | 261.15 | 36.85 | $35.22x10^{-3}$ | $82.50x10^{-3}$ | [1] |
| Benzoic Acid | 122.12 | 122.40 | 40.00 | 5.94 | 19.50 | [2] |
| 2-Naphthol | 144.17 | 122.00 | 40.00 | 1.32 | 6.38 | [2] |
| Salicylic Acid | 138.12 | 158.65 | 40.00 | 3.80 | 8.21 | [2] |
| Glibenclamide | 494.00 | 169.00 | 35.00 | 0.192 | 0.463 | [3] |
| Emtricitabine | 247.24 | 137.00 | 35.05 | $1.16x10^{2}$ | $1.81x10^{2}$ | [4] |
| Oxolinic Acid | 261.23 | 314.00 | 35.00 | $2.3600x10^{-2}$ | $4.8936x10^{-2}$ | [5] |
| Glipizide | 445.54 | 202.00 | 35.05 | $2.60x10^{-2}$ | $4.13x10^{-2}$ | [6] |
| Ketoconazole | 531.43 | 146.00 | 35.05 | $1.55x10^{-2}$ | $8.75x10^{-2}$ | [7] |
| Amygdalin | 457.43 | 225.00 | 37.00 | $1.34x10^{2}$ | $1.36x10^{2}$ | [8] |

For all the cases in which the experimental solubility at 37°C was not available, the closest experimental data point has been reported, together with the GastroPlus™ prediction at that temperature. In the papers in which the solubility was indicated using the molar fraction of the solute, the conversion to grams/liter has been executed by approximating the solution density to 1 kg/L (the same as pure water), using the formula here reported:

$$Sol_{\left(\frac{g}{L}\right)} = \frac{d_{solvent} * x_{solute} * MW_{solute}}{MW_{solvent} * (1 - x_{solute}) + x_{solute} * MW_{solute}}$$

where

$d_{solvent}$ = density of the solution (grams/liter), which we approximate to 1000 g/L

$x_{solute}$ = molar fraction of the solute (the molecule considered)

$MW_{solute}$ = molecular weight of the solute (grams/mol)

$MW_{solvent}$ = molecular weight of the solvent (18 grams/mol, in case of water)

The formula comes from the mathematical adaptation of the following equation:

$$g_{solute,1mol} : g_{solvent,1mol} = g_{solute,1\ liter} : g_{solvent,1\ liter}$$

where

$g_{solute,1mol}$ = weight of the solute in 1 mol of solution (in grams)

$g_{solvent,1mol}$ = weight of the solvent in 1 mol of solution (in grams)

$g_{solute,1\ liter}$ = weight of the solute in 1 liter of solution (in grams)

$g_{solvent,1\ liter}$ = weight of the solvent in 1 liter of solution (in grams)

## References


[1] F. L. Mota, A. P. Carneiro, A. J. Queimada, S. P. Pinho, and E. A. Macedo, "Temperature and solvent effects in the solubility of some pharmaceutical compounds: Measurements and modeling," *Eur J Pharm Sci,* vol. 37, no. 3-4, pp. 499-507, Jun 28 2009.

[2] J. M. P. Q. Delgado, "Experimental data of solubility at different temperatures: a simple technique," *Heat and Mass Transfer,* vol. 43, no. 12, pp. 1311-1316, 2006.

[3] G. A. Shazly, N. Haq, and F. Shakeel, "Solution thermodynamics and solubility prediction of glibenclamide in Transcutol + water co-solvent mixtures at 298.15-333.15 K," *Arch Pharm Res,* vol. 37, no. 6, pp. 746-51, Jun 2014.

[4] F. Shakeel, N. Haq, I. Alsarra, and S. Alshehri, "Solubility Data, Solubility Parameters and Thermodynamic Behavior of an Antiviral Drug Emtricitabine in Different Pure Solvents: Molecular Understanding of Solubility and Dissolution," *Molecules,* vol. 26, no. 3, Jan 31 2021.

[5] A. Jouyban, S. Romero, H. K. Chan, B. J. Clark, and P. Bustamante, "A cosolvency model to predict solubility of drugs at several temperatures from a limited number of solubility measurements," *Chem Pharm Bull (Tokyo),* vol. 50, no. 5, pp. 594-9, May 2002.

[6] M. A. Kalam *et al.*, "Solubility Measurement and Various Solubility Parameters of Glipizide in Different Neat Solvents," *ACS Omega,* vol. 5, no. 3, pp. 1708-1716, Jan 28 2020.

[7] A. Jouyban, W. E. Acree, and F. Martínez, "Dissolution thermodynamics and preferential solvation of ketoconazole in some {ethanol (1) + water (2)} mixtures," *Journal of Molecular Liquids,* vol. 313, 2020.

[8] A. Aydi *et al.*, "Solubility, Solution Thermodynamics, and Preferential Solvation of Amygdalin in Ethanol + Water Solvent Mixtures," *Pharmaceuticals (Basel),* vol. 13, no. 11, Nov 16 2020.
